# Mathematical modelling of the CSF system: effects of microstructures and posture on optic nerve subarachnoid space dynamics

**DOI:** 10.1186/s12987-022-00366-4

**Published:** 2022-08-30

**Authors:** Petter Holmlund, Karen-Helene Støverud, Anders Eklund

**Affiliations:** 1 Department of Radiation Sciences, Radiation Physics, Biomedical Engineering, Umeå University, 901 87 Umeå, Sweden; 2 Department of Health Research, SINTEF Digital, Trondheim, Norway; 3 Umeå Center for Functional Brain Imaging, Umeå University, 901 87 Umeå, Sweden

**Keywords:** CSF dynamics, Optic nerve subarachnoid space, Translaminar pressure, Glaucoma, Posture, Compartmentalization, Ocular glymphatics, Numerical modelling

## Abstract

**Background:**

The pressure difference between the eye and brain in upright postures may be affected by compartmentalization of the optic nerve subarachnoid space (ONSAS). Both pressure and deformation will depend on the microstructures of the ONSAS, and most likely also on ocular glymphatic clearance. Studying these factors could yield important knowledge regarding the translaminar pressure difference, which is suspected to play a role in normal-tension glaucoma.

**Methods:**

A compartment model coupling the ONSAS with the craniospinal CSF system was used to investigate the effects of microstructures on the pressure transfer through the ONSAS during a posture change from supine to upright body postures. ONSAS distensibility was based on MRI measurements. We also included ocular glymphatic flow to investigate how local pressure gradients alter this flow with changes in posture.

**Results:**

A compartmentalization of the ONSAS occurred in the upright posture, with ONSAS porosity (degree of microstructural content) affecting the ONSAS pressure (varying the supine/baseline porosity from 1.0 to 0.75 yielded pressures between − 5.3 and − 2 mmHg). Restricting the minimum computed porosity (occurring in upright postures) to 0.3 prevented compartmentalization, and the ONSAS pressure could equilibrate with subarachnoid space pressure (− 6.5 mmHg) in $\le$ 1 h. The ocular glymphatics analysis predicted that substantial intraocular-CSF flows could occur without substantial changes in the ONSAS pressure. The flow entering the ONSAS in supine position (both from the intraocular system and from the cranial subarachnoid space) exited the ONSAS through the optic nerve sheath, while in upright postures the flow through the ONSAS was redirected towards the cranial subarachnoid space.

**Conclusions:**

Microstructures affect pressure transmission along the ONSAS, potentially contributing to ONSAS compartmentalization in upright postures. Different pathways for ocular glymphatic flow were predicted for different postures.

## Introduction

Severe ocular conditions such as glaucoma and papilledema have been related to abnormal flows and pressures in the cerebrospinal fluid (CSF) within the optic nerve subarachnoid space (ONSAS) [1–5]. The CSF dynamics of the ONSAS are not fully understood, but the CSF communicates between the ONSAS and the intracranial subarachnoid space (SAS), and evidence suggest that CSF is absorbed along the ONSAS through the optic nerve sheath (ONS) to reach the lymphatic system [6–8], providing a CSF absorption route and a possible outlet for waste products [8]. Thus, disturbances in the local flow and pressure dynamics of the ONSAS may contribute to disorders related to both the eye and the brain. Due to the small size and sensitive anatomical location of the ONSAS, it is difficult to perform in vivo pressure and flow measurements in this compartment. Therefore, mathematical models provide an attractive tool for investigating the ONSAS dynamics by predicting the flow and pressure numerically, based on available in vivo data.

Most mathematical models and clinical fluid dynamic investigations of the ONSAS mainly reflect horizontal body positions and in special cases head-down tilt [1, 2, 9–14], not including upright postures. As craniospinal CSF pressures and volumes change between different postures due to gravity [15–17], the pressure and flow in the ONSAS are altered in upright postures compared to horizontal postures. Based on MRI data of the ONSAS, we previously predicted a potential compartmentalization of the ONSAS in the upright posture, hindering flow and pressure transmittance between the eye and brain [18], possibly protecting the eye from low CSF pressures. However, the predictive model used in this previous study did not include the effects of the complex system of microstructures occupying the ONSAS [19]. The ONSAS is traversed by trabeculae, pillars, and septae, which connect the pia around the optic nerve to the arachnoid membrane [20, 21] and may therefore affect flow and pressure in the ONSAS. Kaskar et al. modelled the CSF circulation in the cranial SAS and ONSAS in the supine position and found that the resistance to flow through the ONSAS was heavily dependent on the density of these microstructures [9]. Moreover, they concluded that the ONSAS pressure was highly sensitive to the ONSAS resistance. To fully investigate the ONSAS dynamics, these effects should be included in the predictive modelling.

Furthermore, the findings of a possible glymphatic system for the eye, with CSF entering the ONSAS from the intraocular side and then passing through the optic nerve sheath [8, 22], provide an additional fluid dynamic component that may affect the ONSAS pressure and flows. Investigating the effects of such a pathway in both horizontal and upright postures could contribute valuable insight into ocular glymphatic function and its driving pressure gradients.

Thus, the aim of the current study was to apply mathematical modelling to investigate how ONSAS microstructures affect ONSAS pressure and flow during a change from supine to an upright body posture, thereby further evaluating the previously suggested postural compartmentalization of the ONSAS. A secondary aim was to investigate how these flows and pressures would align with an ocular glymphatic flow pathway. This manuscript will describe the model implementation and showcase results of predicted flows and pressures for the ONSAS, including both temporal changes and steady state levels.

## Methods

The postural model for the *craniospinal* CSF space was first presented by Gehlen et al. [23] building on work by Qvarlander et al. [24] and Magnaes [17]. The ONSAS compartment was subsequently added in Holmlund et al. [18] and in the current study ONSAS microstructures are added through porous media modelling in addition to a CSF pathway from the intraocular system to the ONSAS, reflecting ocular glymphatics [8, 22]. The calculated model variables are listed and described in Table 1. The basal input data (input parameters) to the model can be found in Table 2. After the mathematical model description, a section describing the different tests/simulations is provided.

**Table 1:** List of the variables calculated during the simulations

Variable name	Description
$\Delta {V}_{c}$	CSF volume change cranially, from supine baseline
$\Delta {V}_{s}$	CSF volume change spinally, from supine baseline
$\Delta {V}_{ONSAS}$	CSF volume change in the ONSAS, from supine baseline
${Q}_{ou{t}_{c}}$	CSF outflow, cranially
${Q}_{ou{t}_{s}}$	CSF outflow, spinally
${Q}_{ou{t}_{ONS}}$	CSF outflow across the optic nerve sheath
${Q}_{c-s}$	CSF flow between the cranial and spinal compartment (positive in cranial-spinal direction)
${Q}_{c-ONSAS}$	CSF flow between he cranial compartment and the ONSAS
${Q}_{LC}$	CSF inflow from the eye across the lamina cribrosa
${p}_{c}$	Pressure in the cranial compartment (ICP) Hydrostatic ref: auditory meatus
${p}_{s}$	Pressure in the spinal compartment Hydrostatic ref: venous hydrostatic indifference point
${p}_{ONSAS}$	Pressure in the ONSAS Hydrostatic ref: the lamina cribrosa
${p}_{{c}_{LC}}$	ICP referenced to the hydrostatic level of the lamina cribrosa
${p}_{vc}$	Dural sinus pressure Hydrostatic ref: auditory meatus
${R}_{ONSAS}$	Total flow resistance in the ONSAS
${R}_{LC}$	Resistance to flow across the lamina cribrosa
${h}_{s-c}$	Vertical height from the spinal to the cranial reference points
${h}_{c-lc}$	Vertical height from the cranial reference point to the lamina cribrosa
$\kappa$	Permeability of the ONSAS*
${r}_{ONS}$	Radius of the optic nerve sheath*
${r}_{ON}$	Radius of the optic nerve*
${C}_{ONSAS}$	Total compliance of the ONSAS
$D$	Distensibility of the ONSAS*
$\varphi$	ONSAS porosity*
$\alpha$	Upper body tilt-angle

*Varies along the ONSAS

**Table 2:** This is a list of the parameters for simulations of a healthy subject

Parameter	Notation	Value	Units	Refs.
Spinal venous pressure	${p}_{vs}$	4.2	mmHg	[53]
Viscous pressure loss below the jugular veins	${{p}_{vi}}_{s}$	2.2	mmHg	[46]
Viscous pressure loss above the jugular veins	${{p}_{vi}}_{c}$	2	mmHg	[46]
Orbital pressure	${p}_{orb}$	3	mmHg	[52]
Reference pressure CSF in supine	${p}_{ref}$	9.1	mmHg	[80]
Exponential constant	${p}_{1}$	2.1	mmHg	As in Gehlen et al. [23]. ${p}_{ref}$ and (${p}_{{c}_{sup}}$) are required
Offset pressure spinally	${p}_{0s}$	4.9	mmHg	Equation (14). ${p}_{vs}$ and ${p}_{ref}$ are needed
Offset pressure cranially	${p}_{0c}$	0.7	mmHg	Equation (14). ${p}_{vs}$,${{p}_{vi}}_{s}$, ${{p}_{vi}}_{c}$ and ${p}_{ref}$ are needed
ONSAS pressure at baseline	${p}_{{0}_{ONSAS}}$	7.35	mmHg	Chosen so that ΔV_ONSAS = 0 in supine equilibrium
CSF formation rate	${Q}_{f}$	0.35	ml/min	[29, 81]
Craniospinal outflow resistance	${R}_{out}$	8.6	mmHg/(ml/min)	[33]
Resistance of the ONS	${R}_{ou{t}_{ONS}}$	3968	mmHg/(ml/min)	Calc. from a “permeability” measure assessed by Raykin et al. [34] times ${\overline{A} }_{ONS}$
Resistance of cranial cervical junction	${R}_{CS}$	0.001	mmHg/(ml/min)	[35]
Baseline LC resistance	${R}_{LC}$	1.9e6	mmHg/(ml/min)	See sensitivity analysis. Section “Specific flow resistances”
Density of blood	${\rho }_{b}$	1060	kg/m^3^	–
Density of CSF	${\rho }_{CSF}$	1000	kg/m^3^	–
Viscosity of CSF	$\mu$	0.9	mPa s	–
Gravitational acceleration	$g$	9.81	m/s^2^	–
Distance from jugular vein collapse to auditory meatus	${l}_{ijv-c}$	0.1	m	[53]
Length from venous HIP to the jugular vein collapse	${l}_{s-ijv}$	0.276	m	[53]
Length from venous HIP to the auditory meatus	${l}_{s-am}$	0.376	m	[53]
Cranio-spinal elastance coefficient	$E$	0.2	1/ml	[82]
Constant reflecting spinal compliance contribution in supine	${k}_{s}$	0.35	–	[83, 84]
Relative spinal outflow resistance	${ROF}_{s}$	0.05	–	Estimated as in Gehlen et al. [23]
Distance from auditory meatus to posterior LC	${l}_{AP}$	0.0519	m	[51]
Distance from cornea to anterior side of LC	${l}_{SI}$	0.0379	m	[51]
Average ONS distensibility along the optic nerve*	$D$	0.018	mm/mmHg	$D$ and ${D}_{i}$ are calculated as in Holmlund et al. [18] using ONS sizes from HUT and HDT MRI
Intraocular pressure at the LC	$IOP$	18.9 sup 15.1 upright	mmHg	[51]

*HUT* head-up tilt, *HDT* head-down tilt

*These distensibility values also agree well with that acquired in other MRI studies [85]

### Mathematical model

#### Differential equations for the CSF compartments

The model consists of a system of three ordinary differential equations (ODEs) describing the CSF volume change cranially ($\Delta {V}_{c}$), spinally ($\Delta {V}_{s}$), and in the ONSAS ($\Delta {V}_{ONSAS}$), where $\Delta V$ indicates the deviation in volume from supine equilibrium ($\Delta V=V-{V}_{su{p}_{eq}}$). The ODE system is solved for *t* > *0* and reads:

1 $$\frac{\partial\Delta {V}_{c}}{\partial t}= {Q}_{f}-{Q}_{ou{t}_{c}}-{Q}_{c-s}-{Q}_{c-ONSAS}$$

2 $$\frac{\partial {\Delta V}_{s}}{\partial t}={Q}_{c-s}-{Q}_{{out}_{s}}$$

3 $$\frac{\partial {\Delta V}_{ONSAS}}{\partial t}= {{Q}_{LC}+ Q}_{c-ONSAS}-{Q}_{ou{t}_{ONS}}.$$

**Fig. 1:**
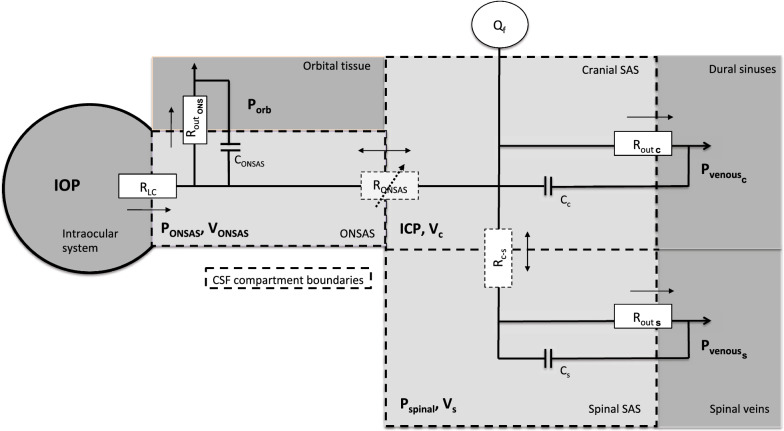
The CSF model. The model consists of three cerebrospinal fluid (CSF) compartments with their own pressure $$p$$, volume $$V$$, and compliance $$C$$. Changes in volume, from supine equilibrium, are denoted $$\Delta V$$. CSF is formed in the cranial CSF compartment (denoted $${Q}_{f}$$) and across the lamina cribrosa (LC) (across $${R}_{LC}$$), and the CSF is absorbed to venous blood (across $${R}_{{out}_{c}}$$ and $${R}_{{out}_{s}}$$, respectively) and across the optic nerve sheath (ONS) (across $${R}_{ou{t}_{ONS}}$$). The spinal ($${p}_{vs}$$) and cranial ($${p}_{vc}$$) venous pressures as well as the intra-orbital pressure ($${p}_{orb})$$ constitute backpressures to CSF absorption and contribute to CSF compliance. Intraocular pressure is denoted as $$IOP$$. In between compartments we have the resistances $${R}_{ONSAS}$$ and $${R}_{C-S}$$ for ONSAS and craniospinal flow, respectively, where the former is dependent on the ONSAS pressure (through MRI—derived ONS distensibility at different sections along the ONSAS [18]). Flow rates $$Q$$ and their directions are indicated with arrows. The model allows for postural changes through hydrostatic effects that alter pressures and redistributes CSF volumes. The collapse of the jugular veins is included for controlling postural changes in the cranial venous pressure, and by extension ICP [23, 24, 53]. The reference level for the $${p}_{c}=ICP$$ is the level of the auditory meatus, the $${p}_{s}$$ is referenced to the venous hydrostatic indifference point (HIP), and $${p}_{ONSAS}$$ to the LC

${Q}_{ou{t}_{c}}$ and ${Q}_{ou{t}_{s}}$ represent cranial and spinal absorption/outflow to venous blood, for example via the arachnoid granulations [25, 26], and ${Q}_{ou{t}_{ONS}}$ the outflow from the ONSAS across the ONS [6, 7]. ${Q}_{c-s}$ is the exchange of fluid between the cranial and spinal SAS (c-s indicates that the positive flow direction is from the cranial towards the spinal compartment) and ${Q}_{c-ONSAS}$ is the fluid exchange between the cranial SAS and the ONSAS, and ${Q}_{f}$ corresponds to the CSF formation (applied cranially) and was assumed to be constant [27]. We also included a source of CSF formation across the lamina cribrosa ($LC$) membrane at the interface between the ONSAS and the eye, ${Q}_{LC}$ [8, 22]. For an overview of the model and its compartments, see Fig. 1.

#### Pressure and flow

The CSF outflow rates are determined by the pressure differences and outflow resistances across the absorption pathways [28–30], and take the form

4 $$Q= \frac{1}{R}\left({p}_{comp}-{p}_{back}\right).$$

The compartmental CSF pressures (${p}_{comp}$) are denoted ${p}_{c}$, ${p}_{s}$ and ${p}_{ONSAS}$, corresponding to the cranial CSF pressure (or ICP), spinal CSF pressure and ONSAS pressure, respectively, while the backpressures for CSF absorption (${p}_{back}$) are denoted ${p}_{vc}$, ${p}_{vs}$, and ${p}_{orb}$ and represent dural sinus pressure, spinal venous pressure, and intra-orbital pressure (the pressure surrounding the ONS) Fig. 1. The hydrostatic reference levels for these pressures are the auditory meatus, the venous hydrostatic indifference point (HIP) and the $LC$, for the cranial, spinal and ONSAS compartment, respectively. The distribution of cranial and spinal absorption were calculated in the same manner as in Gehlen et al., utilizing the total CSF outflow resistance of the craniospinal system (${R}_{out}$) [23]. The outflow across the ONS was defined as:

5 $${Q}_{ou{t}_{ONS}}= \frac{1}{{R}_{ou{t}_{ONS}}}\left({p}_{ONSAS}-{p}_{orb}\right),$$

where ${R}_{ou{t}_{ONS}}$ represents the outflow resistance across the optic nerve sheath (see next sub-section for the definition). All CSF absorption routes are modelled as diodes, i.e., no backflow is allowed [31, 32].

**Fig. 2:**
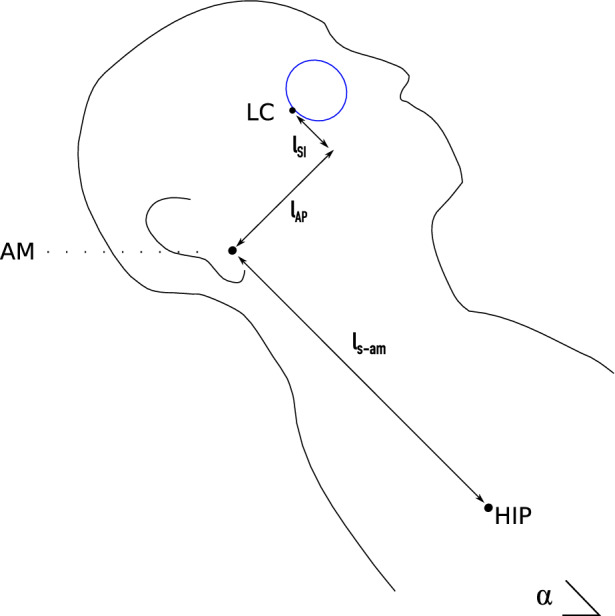
Distances relating the hydrostatic reference points for the CSF compartments. The auditory meatus (AM) corresponds to the reference point for the cranial CSF compartment $(c)$, the venous hydrostatic indifference point (HIP) is the reference for the spinal compartment $(s)$, and the lamina cribrosa $(LC)$ is the reference point for ONSAS. Here the tilt-angle is: $\alpha =45^\circ$. The height from ($s$) to $(LC)$ becomes: ${h}_{s-lc} = {l}_{s-am}\mathrm{sin}\left(\alpha \right)+\left({l}_{AP}\mathrm{cos}\alpha +{l}_{SI}\mathrm{sin}\alpha \right).$

For flows in between different CSF compartments, we used the following equations in the model:

6 $${Q}_{C-S}= \frac{1}{{R}_{CS}}\left({p}_{c}-{(p}_{s}-{\rho }_{CSF}g{h}_{s-c}\right))$$

7 $${Q}_{C-ONSAS}= \frac{1}{{R}_{ONSAS}}\left(({p}_{c}-{\rho }_{CSF}g{h}_{c-lc})-{p}_{ONSAS}\right),$$

where ${R}_{CS}$ is the flow resistance between the cranial and spinal CSF spaces, and ${R}_{ONSAS}$ is the (axial) flow resistance between the cranial SAS and the ONSAS posterior to the eye. The hydrostatic terms on the right-hand side of Eqs. (6–7) are included to account for hydrostatic differences between the reference levels for ${p}_{c}$, ${p}_{s}$ and ${p}_{ONSAS}$. ${\rho }_{CSF}$ represents CSF density, $g$ the gravitational acceleration, and ${h}_{s-c}$ and ${h}_{c-lc}$ are the vertical distances between the different reference points (see Fig. 2).

The inflow across the lamina cribrosa was, in a similar way, set to

8 $${Q}_{LC}= \frac{1}{{R}_{LC}}\left(IOP-{p}_{ONSAS}\right).$$

where $IOP$ is intraocular pressure and ${R}_{LC}$ is the flow resistance across the $LC$.

#### Specific flow resistances

The resistances related to CSF absorption include ${R}_{out}$ and ${R}_{ou{t}_{ONS}}$. ${R}_{out}$ has previously been assessed by in vivo infusion measurements in healthy [33]. ${R}_{ou{t}_{ONS}}$ was estimated from permeability measurements in porcine ONS [34] (Table 2).

For flows in between compartments, we have ${R}_{CS}$ and ${R}_{ONSAS}$. ${R}_{CS}$ was calculated based on the size of the spinal CSF space at the level of foramen magnum [35] (Table 2). Similarly, the ${R}_{ONSAS}$ was calculated based on the size of the ONSAS. We separated the ONSAS into 14 sections, each being 2 mm in length. These 14 sections together cover 4 regions; bulbar, midorbital 1, midorbital 2, and intra-canicular parts of the optic nerve (see Table 3). Subdivision of the ONSAS into several sections is important as the ONSAS decreases in size in upright postures [18, 36], introducing substantial flow resistance in this position. Thus, allowing for variations in size, distensibility and microstructural content along the ONSAS will be crucial for the estimation of this resistance. Assuming that the ONSAS is a porous medium, we may calculate the regional resistances based on Darcy’s law:

**Table 3:** Radii and permeabilities used for calculating the ONSAS resistance at baseline

Region	$\varphi$	${\kappa }_{i}\left(\varphi \right)$(m^2^)	${mean \,r}_{ons}$(mm)	${mean \, r}_{on}$(mm)	R (mmHg/(ml/min))
Bulbar (length 8 mm)	0.75	1.8e−12	2.54	1.75	47.4
	0.85	6.5e−12	2.54	1.75	12.9
	0.9	1.6e−11	2.54	1.75	5.3
	0.95	6.0e−11	2.54	1.75	1.4
	Free flow	N/A	2.54	1.75	9e−4
Mid-orbital 1 (length 8 mm)	0.75	2.0e−11	1.93	1.46	9.2
	0.85	7.2e−11	1.93	1.46	3.2
	0.9	1.7e−10	1.93	1.46	1.3
	0.95	6.7e−10	1.93	1.46	0.3
	Free flow	N/A	1.93	1.46	2.5e−3
Mid-orbital 2 (length 10 mm)	0.75	2.0e−11	1.79	1.49	18.9
	0.85	7.2e−11	1.79	1.49	5.2
	0.9	1.7e−10	1.79	1.49	2.1
	0.95	6.7e−10	1.79	1.49	0.6
	Free flow	N/A	1.79	1.49	6.9e−3
Canicular (length 8 mm)	0.75	3.1e−11	1.78	1.53	11.3
	0.85	1.1e−10	1.78	1.53	3.1
	0.9	2.7e−10	1.78	1.53	1.3
	0.95	1.0e−9	1.78	1.53	0.3
	Free flow	N/A	1.78	1.53	1.1e−3

The radii are here presented as averages over the regions and the resistances are the total resistances over each region. Permeabilities were calculated as in Kaskar et al. [9]

9 $$\left.\begin{array}{c}{R}_{ONSAS}=\sum_{i}{{R}_{ONSAS}}_{i}\\ {R}_{ONSA{S}_{i}}=\frac{\upmu {L}_{i}}{{\kappa }_{i}(\varphi ){A}_{i}},\end{array}\right\}$$

where the subscript $i$ denotes each section of the ONSAS, $\mu$ is the dynamic CSF viscosity, ${L}_{i}$ is the length of the section, $\varphi$ is the porosity, and $A$ is the annular cross section of the ONSAS, i.e., ${A}_{i} =\uppi ({r}_{{ONS}_{i}}^{2}-{r}_{{ON}_{i}}^{2})$ where ${r}_{ON}$ is the radius of the optic nerve and ${r}_{ONS}$ is the radius of the optic nerve sheath. ${\kappa }_{i}(\varphi )$ is permeability along the ONSAS calculated as in Kaskar et al. [9, 37, 38] assuming flow perpendicular to the ONSAS microstructures. The baseline (supine) porosity was altered between *0.95, 0.9, 0.85* and *0.75* in separate simulations to determine its effects on ONSAS dynamics. The first two values are similar to that estimated in the SAS in general [39–41], and introduce only minor effects on the ONSAS pressure in supine. The last two are included to account for the possibility of a denser ONSAS [20]. The baseline radii and permeabilities used for the ONSAS are presented in Table 3. For comparison, we also calculated the resistance based on Poiseuille flow in an annulus, reflecting the case with a negligible resistance contribution from the trabeculae, pillars and septae:

10 $$\left.\begin{array}{c}{R}_{ONSAS}=\sum_{i}{{R}_{ONSAS}}_{i}\\ {R}_{ONSA{S}_{i}}= \frac{8\mu }{\pi {r}_{{ON}_{i}}^{4}}\cdot \left(\frac{{L}_{i}}{{{k}_{i}}^{4}-1-\frac{{\left({{k}_{i}}^{2}-1\right)}^{2}}{ln\left({k}_{i}\right)}}\right)\end{array}\right\}$$

where $${k}_{i}= \frac{{r}_{{ONS}_{i}}}{{r}_{{ON}_{i}}}$$.

${R}_{LC}$ was calculated using the formula in Eq. (9) and the relationship

11 $$\kappa =\frac{\mathrm{K}\mu }{\rho g} \to {R}_{LC}=\frac{L\rho g}{\mathrm{K}A},$$

where values for the hydraulic conductivity $\mathrm{K}$ of the $LC$ could be acquired from Ayyalasomayajula et al. [42] and the thickness/length and area from work by Jonas et al. [43, 44]. There are indications, however, that the flow may mainly pass through specific routes through the $LC$ [8, 22], suggesting a lower resistance than predicted by Eq. (11). For this reason, we performed separate simulations where ${R}_{LC}$ was altered in a sensitivity analysis.

#### Relationship between CSF pressure and volume

As is presented by Gehlen et al. [23], the CSF pressure and volume relationship can be separated into a cranial and spinal part written as:

12 $$\Delta {V}_{c}= \frac{1-{k}_{s}}{E }\cdot \mathrm{ln}\left(\frac{{p}_{c}-{p}_{re{f}_{c}}}{{p}_{1}}\right)$$

13 $$\Delta {V}_{s}= \frac{{k}_{s}}{E }\cdot \mathrm{ln}\left(\frac{{p}_{s}-{p}_{re{f}_{s}}}{{p}_{1}}\right)$$

where the constant ${k}_{s}$ is a measure of the spinal contribution to the total compensatory reserve of the CSF system in the supine position [23], since the reference pressures ${p}_{re{f}_{s}}$ and ${p}_{re{f}_{c}}$ are the same in this position, as are the cranial and spinal CSF pressures, i.e. ${p}_{c}\approx {p}_{s}$ in supine [45].

An important assumption of the original Gehlen model was that the reference pressure was dependent on venous pressure:

14 $${p}_{ref}={p}_{0}+{p}_{v}$$

where ${p}_{v}$ denotes the venous pressure and ${p}_{0}$ is a constant. In the original model, cranial and spinal venous pressures were assumed to be the same in the supine position ${(p}_{vs}={p}_{vc}=CVP)$. However, due to viscous resistance along the veins from the heart to the cranium [46], the cranial and spinal *venous* pressures are somewhat different even in this position, i.e. ${p}_{vs}\ne {p}_{vc}$ [47]. For Eqs. (12–13) to hold, the constant component of the reference pressure $({p}_{0})$ was therefore slightly adjusted cranially to accommodate for this difference, such that ${{p}_{re{f}_{s}}=p}_{0s}+{p}_{vs}={p}_{0c}+{p}_{vc}={p}_{re{f}_{c}}$ in the *supine* position. The viscous resistances were acquired from the literature [46–48].

The relationship between ONSAS volume $\Delta {V}_{ONSAS}$ and pressure ${p}_{ONSAS}$ does not have any established formulation (as opposed to Eqs. (12–13)). From the definition of compliance we can derive an expressionfor this relation:

15 $${C}_{ONSAS}= \frac{\mathrm{d}(\Delta {V}_{ONSAS})}{\mathrm{d}{p}_{ONSAS}}=2\pi {L}_{ONSAS}{r}_{ONS}\frac{d{r}_{ONS}}{dP}=2\pi {L}_{ONSAS}{r}_{ONS}D ,$$

where we assume that the ONSAS is shaped like an annulus. The last factor on the RHS of Eq. (15) reflects the distensibility of the optic nerve sheath:

16 $$D=\frac{d{r}_{ONS}}{dP}.$$

The distensibility was acquired from MRIs of healthy volunteers [18]. Since each of the 13 ONSAS segments each had a unique distensibility (${D}_{i}$), the $D$ in Eq. (15) corresponds to the average distensibility of all ONSAS sections (the 14^th^, or intra-canicular section, was assumed as rigid due to its connection to bone). The distensibility was assumed to be constant for each section (for the investigated pressure range) as the change in volume of the ONSAS is relatively small. The effects of this assumption were investigated by limiting the collapsibility of the ONSAS in a separate analysis (see description below).

#### Postural changes

To model changes in body posture, gravitational effects must be incorporated into the model, which affect both the CSF and the venous system. Within the CSF, hydrostatic effects are already included through Eqs. (6–7). On the venous side, spinal venous pressure in the HIP remains at CVP regardless of body posture, while the cranial venous pressure is affected by hydrostatic effects and the collapse of the jugular veins [47, 49, 50]. The jugular vein collapse is introduced when jugular pressure reaches the surrounding atmospheric zero pressure:

17 $${p}_{jug}=CVP-{\rho }_{b}g\left({h}_{s-ijv}\right)+{p}_{v{i}_{s}}=0.$$

The ${h}_{s-ijv}$ is simply the vertical distance from the HIP (where we have CVP, or ${p}_{vs}$) to the jugular veins, and ${p}_{v{i}_{s}}$ is the viscous resistance from the HIP to the jugulars. This creates a zero pressure reference point on the venous side, and cranial venous pressure can then be calculated by the hydrostatic column from the top of the collapse [47, 49, 50]. It is important to note that the ICP decreases with the cranial venous pressure when moving towards the upright posture [53], as reflected in Eqs. (4 and 12–14).

To allow for compartmentalization of the ONSAS during the posture change, the ONS was allowed to change its size (radius) with changes in pressure

18 $${{r}_{ONS}}_{i}={r}_{ON{S}_{i}}^{Baseline}+{D}_{i}\cdot \left({p}_{ONSAS}-{P}_{Baseline}\right)$$

where $D$ is given by Eq. (16) for each individual segment $i$ (again, except for the intra-canicular segment) and baseline corresponds to the supine position. For the porous media models, the porosity will also change with the pressure since the CSF volume decreases with the pressure. By setting a starting porosity in supine (the model baseline), the cross-sectional area taken up by the microstructures can be determined as

19 $${A}_{ONSA{S}_{i}}\cdot \left(1-{\varphi }_{baseline}\right)={A}_{microst{r}_{i}}$$

and as the ONSAS radius is altered with posture (i.e., pressure), the porosity will change as

20 $$\varphi =1-\frac{{A}_{microst{r}_{i}}}{\pi ({r}_{ON{S}_{i}}^{2}-{r}_{O{N}_{i}}^{2})}$$

where ${{A}_{ONSAS}}_{i}$ is the baseline ONSAS cross-sectional area. This implementation is equivalent to the area of the microstructures remaining unaltered while any changes in ONS/ONSAS size is due to a reduction in CSF space. Since we measured a unique distensibility for each of the 13 segments of the ONSAS (minus the rigid canicular section), the porosity was allowed to vary along the ONSAS (for pressures above or below the baseline).

Finally, the $IOP$ also decreases (slightly) when moving towards an upright posture [51]. This decrease was simply implemented as a linear function of the tilt-angle ($\alpha$). It is unknown if ${p}_{orb}$ changes with posture since in vivo measurements of ${p}_{orb}$ have only been performed in horizontal positions [52]. In the current analysis, we assume a constant ${p}_{orb}$.

### Simulations and set-ups

When running the simulations, the posture change was achieved by linearly increasing the body tilt-angle ($$\alpha$$) from 0° to 90° over the course of 10 s. The posture change was set to occur after 5 min in supine equilibrium, and the simulations continued roughly an hour after the posture change. All CSF flows, volumes and pressures were calculated continuously over time. The simulations correspond to a healthy subject in supine equilibrium with a corresponding ICP ($${p}_{{c}_{sup}}$$) of 11.2 mmHg, which is within the normal range measured in vivo [33, 53–55]. This corresponds to an ICP of 7.35 mmHg at the level of the LC ($${p}_{{c}_{LC}}$$). The $$IOP$$ (at the $$LC$$) started from 18.9 mmHg and was changed to 15.1 mmHg in the upright posture [51]. The simulations were run both with and without the porous media modelling. The baseline porosities tested were 0.95, 0.9, 0.85 and 0.75, where for example 0.75 means that 75% of the ONSAS is occupied by CSF and 25% of microstructures. We also repeated the simulations while imposing limits on the allowed minimum porosity, which is equivalent to limiting the collapsibility (and thus the compartmentalization) of the ONSAS (i.e., a non-collapsible state). The porosity limits tested were 0, 15, 30, 45 and 60%. We also performed a sensitivity analysis regarding the intraocular glymphatic flow by varying the resistance to flow across the $$LC$$ ($${R}_{LC})$$. The resistance was adjusted to allow for an intraocular-ONSAS flow from 0.24% (default) to as much as 24% of the aqueous humour production (the fluid that circulates within the eye), which is about 2.5 $$\mathrm{\mu L}$$ in total [56]. ONSAS pressure, volume, flow, and (minimum) porosity were the main outcome parameters of interest in the analyses.

## Results

### Craniospinal CSF pressures and volumes

**Fig. 3:**
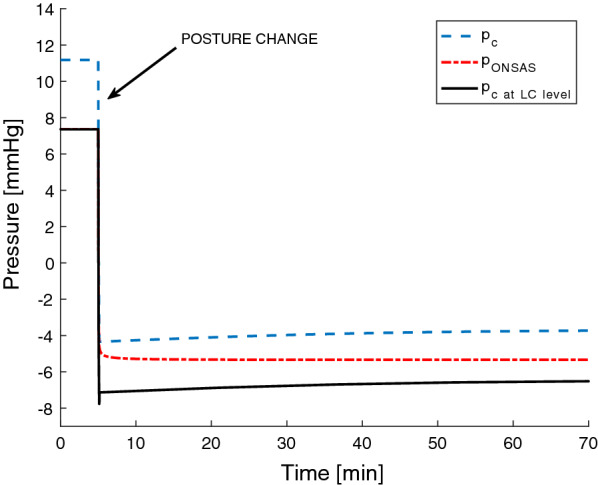
Pressure results for the Poiseuille simulation (i.e., porosity = 1). The ${p}_{c}$ is the ICP at the auditory meatus and ${p}_{{c}_{LC}}$ is the ICP hydrostatically adjusted to the level of the lamina cribrosa, which is the reference level for the ONSAS pressure (${p}_{ONSAS}$). The posture change occurred after 5 min

When moving from supine towards an upright body posture, ICP (${p}_{c}$) decreased and the spinal CSF pressure (${p}_{s}$) increased. After fully standing/sitting up, both pressures increased slightly, due to the CSF formation being slightly larger than the absorption, eventually reaching equilibrium (where CSF formation and absorption are again equal) after about an hour (Fig. 3). At this point, the ${p}_{c}$ had reached − 3.7 mmHg (from 11.2 mmHg at baseline), corresponding to a postural pressure drop that agrees well with reported measurements [24, 53, 55, 57, 58], while the ICP at the level of the LC (${p}_{{c}_{LC}}$) was − 6.5 mmHg (dropping from 7.35 mmHg at baseline) (Fig. 3).

**Fig. 4:**
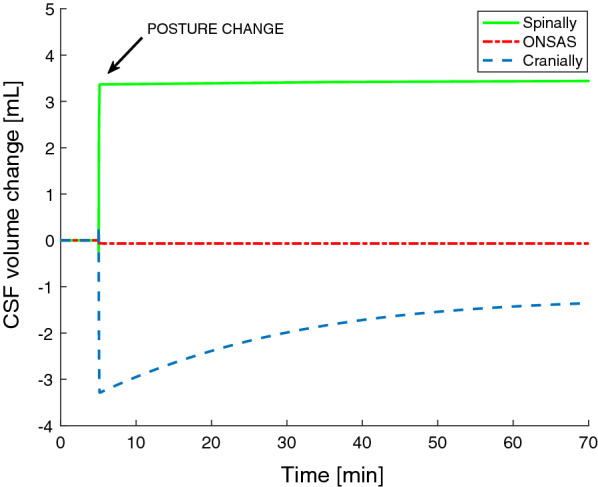
Changes in the compartment volumes for the Poiseuille simulation

The simulated volumetric changes as a function of time are plotted in Fig. 4. Immediately after the posture change roughly 3.3 mL CSF had moved from the cranial to the spinal compartment, which is also in agreement with reported in vivo measurements [17]. From instant upright to steady state, the spinal volume changed only slightly, whereas the cranial volume increased from 3.3 ml below the supine value to 1.2 mL below the supine value, reaching a new equilibrium.

In supine position, absorption/outflow cranially and spinally was 0.31 mL/min and 0.04 mL/min, respectively. In the upright posture, the corresponding numbers were 0.23 mL/min and 0.12 mL/min. From supine to the upright position, ${Q}_{c-s}$ thus increased from 0.04 mL/min to 0.12 mL/min and was always directed from the cranial to the spinal compartment.

### ONSAS pressure and flow

**Fig. 5:**
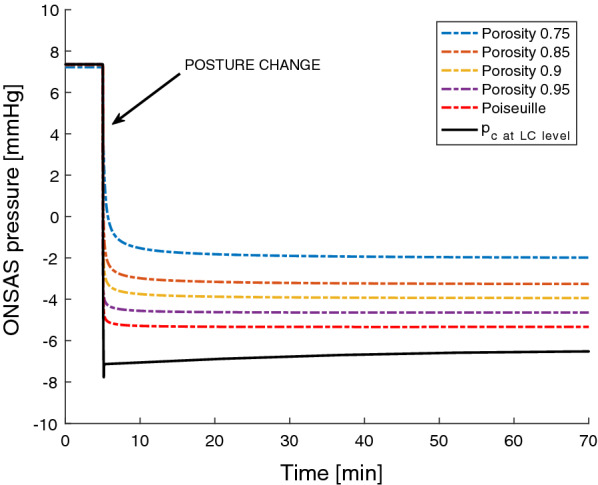
ONSAS pressure during the change in posture for the different porosity settings, showing a strong dependence on the baseline porosity. ONSAS pressures in the upright posture are upheld due to compartmentalization of the ONSAS. The pressures are nearly equilibrated already after 10–15 min

For all simulations, the ONSAS pressure (${p}_{ONSAS}$) decreased when going from the supine to the upright posture due to the postural decrease in ICP (Fig. 5). We start by presenting the results predicted by Poiseuille flow. Until a certain point, ${p}_{ONSAS}$ decreased in a similar fashion as the ${p}_{{c}_{LC}},$ however, the two pressures were eventually decoupled, and the upright pressures differed (− 5.3 mmHg vs. − 6.5 mmHg) (Fig. 3). This was due to ONSAS compartmentalization, as the ONS almost occluded in the midorbital section of the optic nerve (in a posterior slice of the second midorbital region, see Table 3 for the four regions). The corresponding decrease in ONSAS volume was 0.068 mL (Fig. 4). The results for this configuration reflect those in our previous study [18].

**Fig. 6:**
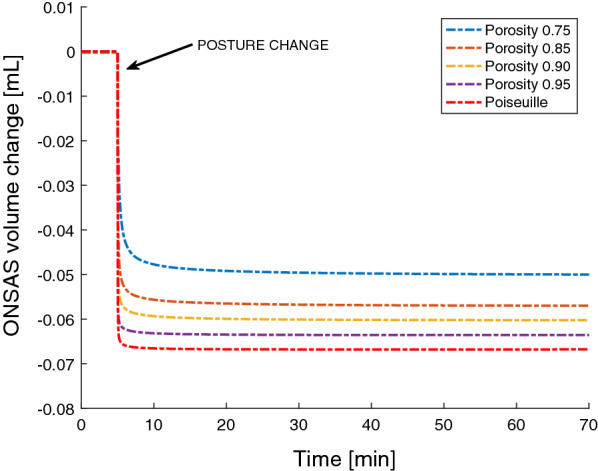
Changes in ONSAS volume when moving form supine to upright for the different baseline porosity settings

Figure 5 displays ${p}_{ONSAS}$ when varying the baseline porosity of the ONSAS, with ${p}_{{c}_{LC}}$ included as a reference (${p}_{ONSAS}$ equals this pressure for low ONSAS flow resistances). All the simulations predicted a compartmentalization of the ONSAS and a break in the communication between ${p}_{ONSAS}$ and ${p}_{{c}_{LC}}$ in the upright posture. The ONSAS pressures reached near-equilibrium within 15 min after the posture change had occurred (Fig. 5). The minimum porosity occurred at the point of ONSAS collapse and varied between 0.14 to 0.06 depending on the assumed porosity in supine (0.95 to 0.75). The baseline porosity clearly affected the equilibrium ${p}_{ONSAS}$. For Poiseuille flow the equilibrium ${p}_{ONSAS}$ was − 5.3 mmHg, for a porosity of 0.9 the ${p}_{ONSAS}$ almost equaled the (midbrain) ICP (− 3.9 vs. − 3.7 mmHg). For the lowest (0.75) porosity setting, ${p}_{ONSAS}$ was upheld to − 2 mmHg. The supine ${p}_{ONSAS}$ slightly decreased with decreasing baseline porosity (Fig. 5). The above-mentioned findings were also reflected in the ONSAS volume (see Fig. 6) where the total change in volume decreased with decreasing baseline porosity (range 67 to 50 $\mathrm{\mu L}$ for the porosity values tested).

**Fig. 7:**
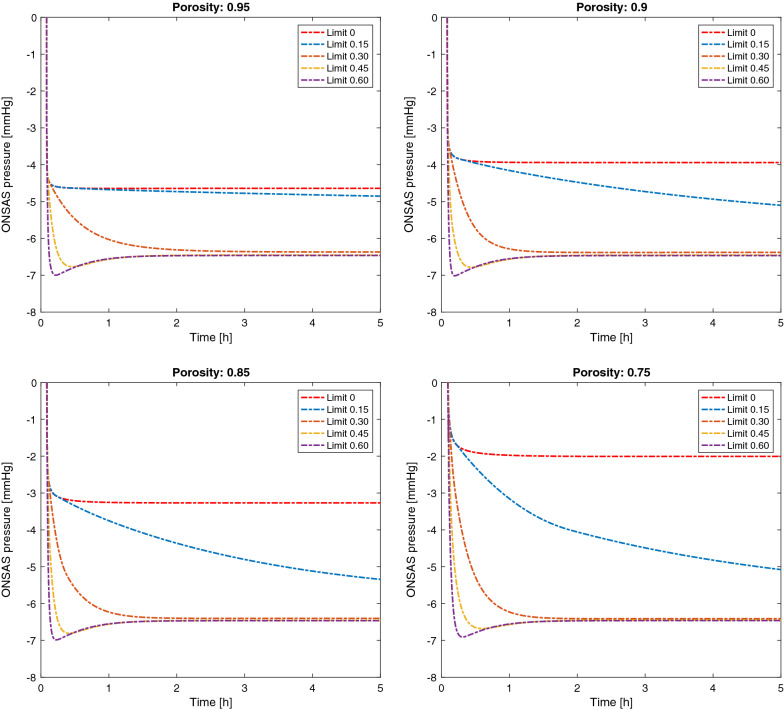
ONSAS pressure for different minimum porosity limits, starting from the baseline porosity noted above each figure panel. A limited porosity hinders complete compartmentalization of the ONSAS in upright postures and the ONSAS pressure equals the ICP at the level of the LC in equilibrium. The dynamic changes are strongly dependent on the porosity limit. NOTE: Time is here in hours, and the image is zoomed in to focus on the dynamics after the posture change (the baseline pressures are not visualized here)

Imposing a lower limit for the calculated porosity introduced a substantial effect on the dynamics of the different models, as well as a lowering of the equilibrium ${p}_{ONSAS}$ since complete compartmentalization was thus not allowed (see Fig. 7). From lower to higher porosity limits there was a transition from a compartmentalized state (with an elevated equilibrium pressure) to a state where the pressure could not be upheld indefinitely and eventually reached that of ${p}_{{c}_{LC}}$. However, for the lowest porosity limit tested (0.15) the decrease in pressure took a very long time (> 5 h) before reaching a state where the two pressures (${p}_{ONSAS}$ and ${p}_{{c}_{LC}}$) were the same. A minimum porosity limit of roughly 0.3 allowed the pressures to equilibrate within an hour upon standing/sitting up. Furthermore, a prohibited compartmentalization allowed for lower pressures to be transmitted along the entire ONSAS, thus, the change in radius close to the $LC$ and the total volume change of the ONSAS was larger for higher porosity limits. Between the minimum (zero) and maximum (60%) porosity limits tested, this increase was roughly 10 $\mathrm{\mu L}$ for baseline porosities 0.95 and 0.9, 15 $\mathrm{\mu L}$ for baseline porosity 0.85 and about 25 $\mathrm{\mu L}$ for baseline porosity 0.75.

The flow rate through the ONSAS in the supine position was close to 0.002 mL/min (0.6% of ${Q}_{f}$) for most simulations and was directed from the cranial SAS to the ONSAS compartment. In the upright position, the equilibrium ${Q}_{ONSAS}$ was  reversed but nearly stagnant (− 1e−5 mL/min). The outflow via the optic nerve sheet (${{Q}_{out}}_{ONS}$) was 0.002 mL/min in supine and zero in upright postures due to the ONSAS pressure dropping below the intraorbital pressure (implemented as a diode).

### Intraocular to ONSAS glymphatic flow

The ocular glymphatic flow across the lamina cribrosa (*LC*) was very low for the calculated ${R}_{LC}$, roughly 6e−6 mL/min in supine and 10e-6 mL/min in upright (0.24% and 0.43% of the aqueous humour production, respectively). The difference between the two values was due to the decreasing ${p}_{ONSAS}$ during the change in posture, as the pressure difference ${IOP- p}_{ONSAS}$ and the resulting glymphatic flow then increased, which is in line with the literature [8]. For all simulations, the flow through the $LC$ passed directly across the ONS in the supine position while it instead flowed along the ONSAS to reach the cranial SAS in the upright posture. Thus, the porosity (or resistance) of the ONSAS mainly affects the glymphatic flow in upright postures. The sensitivity analysis for the intraocular-ONSAS flow revealed that for the lowest tested resistance ${R}_{LC}/100,$ the ONSAS pressure barely changed (from that of the default simulation) in supine, and by less than 1 mmHg in upright. For example, in upright, the above-mentioned setting increased ${p}_{ONSAS}$ by 0.3 mmHg for Poiseuille flow (compared to the default simulation), and by 0.73 mmHg for the 0.75 baseline porosity simulation. The corresponding flow rate was 24% of aqueous humour production in supine and 42% in upright for Poiseuille flow while the change was slightly smaller for the 0.75 baseline porosity simulation, 24% to 34%, due to a higher ONSAS pressure in upright for the porous media model. The full sensitivity analysis is displayed in Table 4.

**Table 4:** Sensitivity analysis for ${R}_{LC}$

Baseline porosity (%)	${Q}_{LC}/{Q}_{for{m}_{Aqueous Humor}}$(%) in supine	${Q}_{LC}/{Q}_{for{m}_{Aqueous Humor}}$(%) in upright posture	${p}_{ONSAS}$ in the upright posture (mmHg)
Poiseuille	0.24 (Default)	0.43	− 5.3
	5	9	− 5.2
	15	27	− 5.1
	24	42	− 5.0
0.95	0.24	0.42	− 4.6
	5	9	− 4.5
	15	26	− 4.4
	24	41	− 4.3
0.9	0.24	0.40	− 3.9
	5	8	− 3.7
	15	25	− 3.5
	24	39	− 3.4
0.85	0.24	0.39	− 3.3
	5	8	− 3.0
	15	24	− 2.8
	24	37	− 2.7
0.75	0.24	0.36	− 2.0
	5	7	− 1.7
	15	22	− 1.4
	24	34	− 1.3

The ${R}_{LC}$ was adjusted to allow for specific supine flow rates ${Q}_{LC}$. Resulting upright flow rates are also presented

## Discussion

To date, the ONSAS pressure and flow alterations between horizontal and upright body postures remain essentially unexplored. Based on existing mathematical models, we developed a new model that allows for prediction of the ONSAS dynamics as a function of body posture including the effects of the microstructures occupying the ONSAS. As the ONSAS is part of the CSF system, a crucial aspect of the model is the interaction between the ONSAS and the craniospinal SAS. This interaction was affected by the content of the ONSAS, possibly allowing for a compartmentalization of the ONSAS in upright postures that introduces significant alterations in steady state ONSAS pressures. The intraocular glymphatic flow increased in upright compared to the supine position, but the flow was redirected from the ONS towards the cranial SAS. Sustaining the glymphatic flow did not require any larger changes in ONSAS pressure even for relatively large intraocular-CSF flows in any body posture.

In clinical studies, the ICP is often used as a surrogate for the ONSAS pressure (${p}_{ONSAS}$) when estimating potential pressure disturbances between the eye and brain [59–61]. Furthermore, the size of the ONSAS posterior to the globe has for some years been investigated as a potential non-invasive indicator of ICP made accessible by ultrasound or MRI [62, 63]. These investigations are based on the assumption of a fully communicating ONSAS and a negligible flow resistance along this pathway. While these assumptions may hold under certain circumstances, e.g., in horizontal body postures (in healthy subjects) or for elevated pressures, they may not hold for all situations. Furthermore, the postural dependency of ICP is often completely overlooked, as the ICP is seldom assessed in upright body postures [51, 58]. Our results open for the possibility that ${p}_{{c}_{LC}}$ and ${p}_{ONSAS}$ are not always the same in upright postures, as even the prediction using Poiseuille flow estimated a small (1 mmHg) difference between the two pressures (Fig. 3). The dependency on ONSAS porosity could mean that differences in porosity, e.g., between different persons or groups of people, could lead to different pressures and flows in the ONSAS (Fig. 5). The model results indicate that a baseline porosity of 0.9 is needed for the ${p}_{ONSAS}$ to mimic midbrain ICP (the common reference for CSF pressure), although the temporal behavior of these pressures differ somewhat (Fig. 5). Thus, if the baseline porosity lies somewhere close to 0.9, midbrain ICP may be a reasonable surrogate for the ONSAS pressure even in upright postures.

One main assumption in our default model [18] is that we extrapolate the distensibility results from 13 to 90 degrees. Imposing a lower limit on the calculated porosity is a way to remedy this, and to investigate the effects of this assumption (Fig. 7). The results show that the limit must be low (roughly 0.1) to uphold a pressure above ${p}_{{c}_{LC}}$ indefinitely, while a limit in between 0.3 and 0.1 may keep the pressure elevated in between one to several hours. Both these options would require a high flow resistance and a low flow in the ONSAS. CT contrast infusions [64] in porcine models has indicated a limited and posture (or gravity) dependent filling of the ONSAS, and could support a limited flow rate in upright postures. If the minimum porosity is higher than 0.3, we can expect relatively quick changes in ONSAS pressure (roughly less than 30 min–1 h to reach ${p}_{{c}_{LC}}$). Another result of imposing a lower limit on the porosity is that the size of the ONSAS keeps decreasing closer to the bulbar region all the way to the 90-degree upright posture, something that is prevented if “complete” compartmentalization occurs. Thus, a larger change in the bulbar ONSAS size may indicate a lack of compartmentalization and a transfer of lower pressures to the back of the eye, or, alternatively, an increased distensibility of the optic nerve sheath.

In the supine position, our simulations predicted a ${p}_{ONSAS}$ equal to the ICP at the level of the $LC$ (that is, ${p}_{{c}_{LC}}$) or slightly lower (Fig. 5). While data are sparse, measurements in animals have shown horizontal ${p}_{ONSAS}$ values similar to ${p}_{{c}_{LC}}$ [65] or values a few mmHg lower [66], and measurements in cadavers yielded a ${p}_{ONSAS}$ in the range of 0–6 mmHg [67]. While the latter may not represent the physiological situation, the animal studies support that non-fluid content within the ONSAS may contribute to a non-negligible resistance already in horizontal positions. Thus, a decreased ONSAS porosity leads to a decreased ONSAS pressure in supine position, but also to an increased ONSAS pressure in upright postures, diminishing the postural effect on the ONSAS pressure in two different ways (Fig. 5).

Our model can be compared to the work by Kaskar et al. who modeled the CSF system in the supine position. Based on the pressure measurements in the cadavers [67], their model predicted an ONSAS resistance of 200–241 mmHg/(mL/min) and an ONS absorption of 5–10% of the total CSF outflow [9]. Their outflow rates are high compared to ours (roughly 5–10% vs. 0.5–0.6%), which may indicate that our ${R}_{ou{t}_{ONS}}$ was overestimated. Decreasing our ${R}_{ou{t}_{ONS}}$ by a factor of 10 would yield a lowered supine ${p}_{ONSAS}$ since the flow rate would then increase, but only for a porosity of 0.75 would the change be larger than 1 mmHg (1.2 mmHg for 0.75, 0.36 mmHg for 0.85, and < 0.15 mmHg for the rest). Since ${p}_{ONSAS}$ quickly dropped below the intra-orbital pressure during the posture change, stopping any ONS absorption, any error in ${R}_{ou{t}_{ONS}}$ will have a limited effect on the dynamic changes during the alteration in posture (Fig. 5). A limitation of the Kaskar model is that the ONS absorption is set as a constant and is not pressure-driven, i.e., it is independent of factors such as the intra-orbital pressure and does not explicitly include any resistance to absorption across the ONS. Because ${p}_{ONSAS}$ is affected by both ${R}_{ONSAS}$ and ${R}_{ou{t}_{ONS}}$, our model does allow for analyses of these contributions separately (in addition to their postural dependency).

### Clinical applications

The ONSAS dynamics are believed to relate to ocular disorders through the trans-lamina cribrosa pressure difference, i.e., the difference between intraocular pressure and ONSAS pressure. A relevant example is normal tension glaucoma where a low ICP has been suspected as a contributing factor to the development of the disorder [5, 59, 68, 69]. Understanding the link between ICP and the pressure within the ONSAS is crucial to accurately interpret findings related to this pressure difference, especially since the trans-LC pressure difference is often just calculated as IOP-ICP, which likely does not hold for all postures (Figs. 3, 5). For example, a lack of compartmentalization could allow for lower pressures to reach the posterior eye in upright postures, thus increasing the pressure difference despite normal IOP and ICP [70]. The ONSAS dynamics may also be disturbed in other ways. For example, there is evidence of a hindered ONSAS flow both in patients with normal-tension glaucoma [1, 71] and those with papilledema [2] in horizontal positions, which could mean that the ONSAS resistance is instead increased in these patients.

Posture, and thus gravitational effects, is also of importance for understanding the spaceflight associated neuro-ocular syndrome, a syndrome where astronauts lose their visual acuity after extended visits in microgravity [72]. An abnormal ICP (and by extension an abnormal ONSAS pressure) is one suggested hypothesis [51, 73, 74] and compartmentalization of the ONSAS another [72, 73]. The pressure behaviour as studied with our model may contribute valuable reference data for comparison to the microgravity state. While ICP measurements during long-duration spaceflight are non-existent, measurements in acute microgravity suggest a decrease in both ICP and CVP by roughly 3–4 mmHg [74]. By removing gravitational effects and lowering the CVP accordingly (3.5 mmHg), our model predicts an ONSAS pressure of 7.7 mmHg in microgravity. This is slightly larger than the supine baseline value of 7.35 mmHg. This happens because the hydrostatic difference between the LC and the auditory meatus disappears in microgravity, and this hydrostatic effect is slightly larger than the microgravity-induced change in CVP/ICP. Furthermore, microgravity removes the normal lowering of ICP that occurs in upright postures on earth, resulting in an, on average, higher ICP in microgravity (assuming 16 h per day are spent upright). The difference in this average will depend on the porosity of the microstructures, where a denser (low porosity) ONSAS will lead to a smaller postural effect on the ONSAS pressure on earth.

The extended model could potentially be used for understanding the clearance of eye metabolites, through the ocular glymphatic system [75] of the ONSAS pathway [8]. Assessing local pressure gradients that drive the fluid flow will be essential for increasing our understanding of this clearance system. The sensitivity analysis of the LC flow resistance (Table 4) revealed that flow from the intraocular system could be quite substantial (even up to 24%) without requiring any larger (< 1 mmHg) changes in the ONSAS pressure, despite the high resistance pathway of the ONSAS in the upright posture. As shown by Wang et al. [8], the flow through the posterior of the eye to the ONSAS depends on the $IOP-{p}_{ONSAS}$ pressure difference (by design in our model). This suggests that flow from the intraocular system increases in upright postures. This is in contrast to glymphatic function in general, which has been shown to be increased during sleep (when we are horizontal) [76]. Our model does predict that clearance across the ONS mainly occurs in supine (horizontal) positions however, while in upright positions the flow is directed to the rest of the SAS, suggesting different pathways for the two postures. Thus, intraocular fluid is absorbed in supine while it contributes to CSF production in upright. If absorption across the ONS still occurs in upright postures, it would likely indicate that the counter pressure to this absorption, i.e., the intraorbital pressure, must change in a similar fashion as ${p}_{ONSAS}$. However, we consider this unlikely as the intraorbital pressure can be suspected to remain fairly constant. An alternative is that some other pressure constitutes the counter pressure to this absorption.

Finally, additional CSF compartments can subsequently be added to the current model, moving towards a more complete description of the CSF dynamics and glymphatic functions. As CSF dynamics do vary with body position, including the effects of posture in such models are likely essential. Additional outflow/absorption routes related to the glymphatic system could be included. However, this should not affect the dynamics investigated in this study as absorption in our model is based on the total outflow resistance ${R}_{out}$.

### Limitations

We extrapolated the distensibility values from 13 degrees head-up tilt (maximum achievable in our scanner) to 90 degrees head-up tilt. Imaging in sitting MRIs could contribute important data here, however, in this study, this limitation was instead addressed by the porosity limitation analysis. We did not include any pulsations in this study, including general CSF pulsations as well as local arterial pulsations in the optic nerve and ONS, which may contribute to the mixing and distribution of CSF between the ONSAS and cranial SAS. The relative contribution from the spinal and cranial compartments to CSF compliance has been debated, where some estimations indicate a larger spinal contribution compared to the cranial contribution [17, 77]. Changing this parameter in this way will lower ICP slightly, exacerbating the effect of the compartmentalization. However, this will increase the postural shift in volume to values much higher than that measured in vivo, as opposed to the current setup, suggesting that our choice recreates the in vivo situation satisfactorily. Only CSF absorption to venous blood was included for the craniospinal CSF compartments despite lymphatic outflow existing for these compartments as well [78, 79]. Since accurate postural craniospinal CSF pressures and volume changes were achieved with the current model, they were deemed sufficient for the specific application of the current study (where we focus on the ONSAS), but these other flow pathways need to be added to study for example general glymphatic flow throughout the brain. There is a lack of studies of ocular glymphatics in the upright posture to validate our glymphatics flow predictions. While our model can predict the outcome of different scenarios, more work in this area is needed to validate these predictions and for further improving the model.

## Conclusions

The current study presents a model for analyzing the ONSAS fluid dynamics and its interactions with the craniospinal CSF spaces with varying body posture. The porosity related to microstructures within the ONSAS was predicted to affect the ONSAS dynamics during the change in posture from supine to upright as well as the equilibrium ONSAS pressure in upright postures. The results thus suggest that measured ICP may not be an appropriate surrogate for the ONSAS pressure in upright postures. While the current study focused on the ONSAS and the analysis of ocular glymphatics, the model can be expanded to include other CSF compartments, offering a tool for gaining a more complete description of the CSF system and its posture dependency.

## Data Availability

No original datasets were used in the present study. All parameters used in the model are described in the methods section and relevant figures.

## Abbreviations

CSF: Cerebrospinal fluid
HDT: Head-down tilt
HUT: Head-up tilt
HIP: Hydrostatic indifference point
ICP: Intracranial pressure
IOP: Intraocular pressure
LC: Lamina cribrosa
MRI: Magnetic resonance imaging
ODE: Ordinary differential equation
ON: Optic nerve
ONS: Optic nerve sheath
ONSAS: Optic nerve subarachnoid space
